# Flames of transformation: Igniting better mental and physical health for racialized and gendered North Americans

**DOI:** 10.3389/fgwh.2023.1126934

**Published:** 2023-02-13

**Authors:** Farah Mahrukh Coomi Shroff

**Affiliations:** ^1^ Department of Family Practice and School of Population and Public Health, Faculty of Medicine, University of British Columbia, Vancouver, BC, Canada; ^2^ Maternal and Infant Health Canada, Vancouver, BC, Canada

**Keywords:** black, indigenous, women of color, mental health, social determinants of health, policy and guidelines

## Abstract

COVID-19 is catalyzing both crises and opportunities for communities of color.^1^ The crisis of high mental and physical morbidities and mortalities exposes persistent inequities while providing opportunities to celebrate the power of rejuvenated anti-racism movements, fueled partly in response to the extremism of ultra-conservative governments, the circumstances to reflect deeply on racism because of forced stay-at-home-orders, and digital technologies primarily driven by youth. In marking this historical moment of longstanding anti-racism and decolonial struggles, I assert the importance of foregrounding women’s needs. In analyzing racism, rooted in colonialism and white supremacy, and its impacts on mental and physical health status, I focus on improving racialized women’s lives within the larger context, concentrating on the determinants of health. I contend that fanning the flames to scathe the racist and sexist foundations of North American society will break new ground for sharing wealth, bolstering solidarity and sisterhood, and ultimately improving Black, Indigenous, and Women of Color (BIWOC) health. Canadian BIWOC earn approximately 59 cents to the dollar earned by non-racialized men, creating vulnerabilities to economic downturns, such as the one Canada is currently in. BIWOC care aides, at the bottom of the healthcare hierarchy, are emblematic of other Black, Indigenous, and People of Color (BIPOC), who face risks of frontline work, low wages, poor job security, unpaid sick days and so forth. To that end, policy recommendations include employment equity initiatives that hire groups of racialized women who consciously express solidarity with each other. Cultural shifts within institutions will be key to providing safe environments. Improving food security, internet access and BIWOC-related data collection linked to community-based programming while prioritizing research on BIWOC will go a long way toward improving BIWOC health. Addressing racism and sexism within the healthcare system, aiming for equitable diagnostic and treatment foci, will require transformative efforts including determined leadership and buy-in from all levels of staff, long-term training and evaluation programs, audited by BIPOC communities.

## Introduction: the spark that lit the bonfire

The edifice of North American society is shifting as a result of decades of anti-racism organizing. The Edmonton Eskimos, Eskimo Pies, the Cleveland Indians and other large corporations, despite persistent refusals to adjust their names, did just that in 2020. One of the unexpected developments during COVID-19 was the flourishing of anti-racism movements and large-scale attitudinal shifts that propelled these changes. Moral persuasion alone was not enough to promulgate these advancements. Threats of lawsuits and lost revenue were the fuel that set fire to the symbolism evoked by these names. While much more needs to be done, this era holds promise for genuine transformation.

The murders of Black, Indigenous, and People of Color (BIPOC) in Canada and the US, while not new, sparked renewed activism all over the continent. With most people at home, facilitating time to reflect deeply on these ongoing deaths, action followed. These renewed anti-racism movements, partially a response to the ultra-conservative governments around the world, were fueled by digital technologies, often driven by youth, and a more diffuse leadership than previous movements. Building on decades-long endeavors for human rights in this continent, the post-2020 anti-racism movements are producing results in some of the most promising quarters–institutions and corporations. Extra efforts to support Black, Indigenous Women of Color (BIWOC) are required.

In this paper, I describe and analyze COVID-19’s impacts on racialized communities in Canada, with a focus on women’s mental and physical health. Canada collects data on Indigenous communities but not always on other racialized communities. Long-term robust Canadian data, disaggregated by ethnocultural status for non-Indigenous populations would make a positive impact on the creation of policies and programs for BIPOC. Nonetheless, based on reasonably solid data from other jurisdictions and similar socioeconomic conditions within Canada, there is enough evidence to paint a picture of compromised health based on structural inequities (1). I argue that racialized women’s mental and physical health issues have been relegated to the shadows and now is the time to reverse this trajectory. This anti-racism moment is moving BIPOC out of the shadows, but the pandemic has rolled back women’s rights and thus efforts to improve BIWOC health require strong social movements and supportive governments and businesses.

## Racism related to the pandemic

BIPOC Canadians, particularly those of Asian descent, have faced pandemic-specific racism including more verbal and physical abuse (2), highlighted by the gruesome murders of American-Asian women in March 2021; anti-racism movements have acted.

Using an intersectional analysis, racism and other structural inequities have created a toxic impact on health status. Inequities related to income and social status—racism, sexism, heterosexism, ableism, ageism and so forth—have borne themselves out in unemployment figures, job insecurities, as well as COVID-19 illnesses and deaths. Carrying out frontline, *essential work*, which is high risk, combined with high-density housing and other factors related to health status, make BIPOC communities at higher risk.

Virtually all racialized groups experienced more difficulty meeting their essential needs during the pandemic than those of European heritage (3). Given that pre-existing poverty rates were higher among BIPOC Canadians than their European heritage counterparts, work disruptions have created more hardship.

## Health impacts of racism on BIPOC communities

Those parts of Canada with the highest proportion (25% or more) of racialized communities experience COVID-19 mortalities at twice the rate of those areas with the lowest proportion of racialized communities (less than 1%). The cosmopolitan metropolises of Toronto and Montreal showed higher death rates in Black communities than in those of European heritage, while Indigenous communities, experiencing high rates of chronic conditions and disabilities, continue to have disproportionately high rates of COVID-19 (4).

Health inequities related to ethnocultural status are based on pre-existing social determinants of Health (SDOH) and underlying health conditions which increase the severity of COVID-19 illnesses for racialized communities, conditional on exposure to the virus (5). A variety of factors are associated with greater ill-health experienced by BIPOC communities:

• Safety concerns related to violence against those who identify as women
• Police brutality
• Greater likelihood of experiencing harassment, attacks, and stigma—hate crimes (6)
• Social and economic determinants of health, particularly front-line work
• Stress caused by racism and other forms of oppression
• Housing density
• Inequitable access to healthcare and social services (7)

Racism produces damaging impacts on mental and physical health status, including depression, suicide, anxiety, Post-Traumatic Stress Disorder (PTSD), drug addiction, feelings of helplessness or worthlessness, avoidance behaviors, hypertension, and increased abdominal adiposity (8). *Weathering, a* premature decline in health status related to racism and other forms of oppression, has been found to reduce life expectancy by 6.1 years in those of African heritage compared to their Euro-heritage counterparts (9).

Inflaming this situation, racism in healthcare creates mistrust, particularly among Indigenous communities, who experience higher morbidity and mortality within treatment settings (10). Joyce Echaquan, an Indigenous mother of seven, did not survive hospitalization. Racism and prejudice “certainly contributed to her death” according to the coroner’s report, as she “was quickly labeled a drug addict and, based on this prejudice, it follows that her cries for help were unfortunately not taken seriously” (11).

### Mental health impacts in BIPOC communities related to the pandemic

In pre-pandemic times, 68% of Canadians reported excellent or very good mental health status. Early in the pandemic, this percentage declined to 55% (12). Consistent with pre-pandemic mental health discrepancies, 52% of women reported lower levels of mental health status compared to 58% of men, while racialized Canadians reported higher levels of anxiety (30%) than those of European heritage (24%) (12).

The impacts of weathering are most widely documented for depression (9). Racism, in combination with poverty, police violence, and other socioeconomic factors, is responsible for disparate mental health outcomes for BIPOC communities (13). Black communities in the USA exhibited higher rates of substance abuse and suicidal ideation during the pandemic than prior to it (14). Similarly, Latinx communities experienced more anxiety and depressive symptoms, along with trauma and stress related disorders, increased substance use, and suicidal ideation during the pandemic, than prior to it (15).

In Canada, racism is strongly mediated by individual occupation, the healthcare system, and living conditions within dwellings and communities at large (16). Gender plays a key factor as BIWOC are overrepresented in frontline work, which presents a double jeopardy of poor pay combined with a high risk of exposure to pathogens (17). Continuing pre-pandemic trends, South Asian Canadians, in particular, reported poor levels of mental wellbeing since physical distancing measures were established, while those of Latinx and Chinese heritage, reported significant decreases in mental wellbeing from 2020 to 2022 (18). In Quebec, Arab and Black communities reported high levels of psychological distress related to viral exposure, COVID-19 related discrimination and mental health stigma (19). Various studies have noted that racialized women’s mental wellbeing has suffered tremendously during the pandemic (20, 21). A Saskatchewan study similarly found that women, aged 30–49 years, who identified as immigrants, earned less than $20,000 per year, and had no or low levels of post-secondary education, struggled more than all other participants with anxiety and depression during the pandemic (22).

Studies of Indigenous Canadians paint a mental health picture that stretches back hundreds of years to enduring vitality and resiliency despite colonialism, genocide, and contact with Europeans. Intergenerational trauma, violence, addictions, and other mental health conditions are linked to racism and colonialism (23). Some Indigenous families had to choose between paying rent and staying at home to care for children (16) despite extra government financial assistance during the pandemic. Extra caregiving responsibilities added to stress levels. Accessing healthcare became difficult during the pandemic as healthcare systems diverted most of their resources to addressing COVID-19. Some Indigenous women reported difficulty with both online and in-person access to care, noting that phone appointments were particularly difficult as participants experienced feelings of awkwardness (16). Indigenous communities in Australia experienced similar challenges, expressing difficulties adapting to the rapid pace of change to telemedicine, combined with the lack of preparation offered by care providers (24). Economic stresses, loneliness, and school closures, in conjunction with longstanding daily realities rooted in marginalization, prompted some Indigenous women to lean on new coping mechanisms such as cannabis, tobacco, alcohol, and prescription medication (16).

Similar to other parts of the world, consumption of substances, such as alcohol (16%), cannabis (6%), and tobacco (5%), increased in Canada during the pandemic across all gender categories (12). Addictions to illicit drugs also increased substantially, with record-breaking fentanyl-related deaths (25) far surpassing COVID-19 related deaths (26).

While addictions increased in all gender categories, women suffered more mental health problems than men, both prior to and during the pandemic. This is largely related to caring, cleaning, and cooking tasks that are gendered, and this extends to youth (27). Women in perinatal stages were at greater risk of mental health concerns during the pandemic than prior to the pandemic (28). Birthing alone, without a spouse or loved ones present, surrounded by care providers in personal protective equipment (PPE), coupled with fears of maternal or infant viral infection, was traumatic for many women (29). Perinatal education programs were also negatively impacted during the pandemic, leaving birthing couples less prepared (30). Pandemic parenting, particularly for mothers, who bear the largest load of caregiving, was often done in isolation from supportive families and others. Parents reported feelings of anxiety, agitation, fear, or sadness related to limited financial and social resources, employment and income challenges, increased addictions and a deep sense of the unknown (28).

Related to long term mental health concerns, the public health pandemic of violence against women increased all over the world. Canada was no exception, with rates approximately double those of pre-pandemic times (31). Women survivors struggle with depression, substance use, and PTSD (32).

The pandemic of violence against women healthcare providers also increased during the pandemic. Globally, women healthcare workers (HCWs) demonstrated negative outcomes related to stress, burnout, and depression during the pandemic. Exacerbating factors at the individual level included lack of social support; women who were single or lacked social support were at greater risk while those with kids were at lower risk. Structural factors included high workloads, shifting public health policies and lack of recognition and PPE (33).

Burnout has been a longstanding concern amongst physicians. Early in the pandemic, Canadian women internal medicine doctors reported higher rates of burnout based on emotional exhaustion, depersonalization, and feelings of low personal accomplishment than their male counterparts. Similarly, racialized physicians reported higher rates of low personal accomplishment than their colleagues of European heritage, making racialized women physicians hardest hit by pandemic stressors. Approximately one of five participants disclosed thoughts of leaving the profession or quitting a position. Of these relatively well-paid and respected HCWs, those who identified as women or ethnic minorities, who had been impacted by COVID-19, experienced higher rates of burnout (34).

Pre-pandemic mental health needs of racialized women in North America were very serious, and yet largely unmet. During the pandemic, unimaginably stressful conditions such as extreme violence in the home and workplace, financial burdens, loneliness, and isolation from social supports, exacerbated virtually every social determinant of mental health. We are now in a severe crisis in relation to the mental health status of BIWOC in North America.

## Health issues facing BIWOC

Scant attention is paid to physical and mental health concerns of BIWOC. Disparities, however, are striking, despite BIWOC buoyancy, strength, and resilience. The cumulative effects of stress are quantified by allostatic load, which measures physiological dysregulation related to long term chronic stress in the body (35), telomere length, which measures longevity amongst other things (36), and other tests. These measurements reveal patterns of poor mental and physical health related to the toxic nature of uncontrollable or unpredictable stressors (37). Long term stressors decrease BIWOC’s coping mechanisms, simultaneously increasing susceptibility to mental health conditions (38).

### Mental health issues amongst BIWOC

Weathering’s mental health impacts are becoming more evident as research progresses in this area. Depression has been identified as one of the biggest impacts of weathering. Everyday discrimination is associated with higher rates of depression for people of African heritage in the US (39).

Microinsults, microassaults, microinvalidations, and other forms of microaggressions on a long term basis create various forms of fear-based responses (40). Studies of African heritage people in the US have found that traumatic life incidences contribute to mental health imbalances; Black children in the US are three times as likely to lose a mother before the age of 10, while Black adults are twice as likely to lose a child before the age of 30 and a spouse before the age of 60 than their counterparts of European heritage (41). Language barriers and challenges with schools and other institutions, compound these traumatic events to create conditions that are ripe for mental illnesses (42). Everyday discrimination puts people of Asian and Latin American heritage at higher risk of both anxiety and depression (43).

The most serious sequela of depression is suicide. Indigenous communities in North America have extremely high rates of suicide: Inuit youth die by suicide at a rate eleven times that of non-Indigenous Canadians (44). Suicide is the second leading cause of death in Indigenous people between the ages of 10 and 34 (45). Suicide is the third leading cause of death within Black communities in the US, between the ages of 15 and 24 (46).

In virtually all societies, women are more likely than men to experience mental health disorders (47). Explanatory theories for this gendered reality are incomplete, but there is some consensus that most societies raise girls to be nurturing, caring, and emotionally expressive, so that when mental health challenges exist, girls and women express their difficulties. Domestic violence, sexual assault, workplace sexual harassment, lower incomes, and other forms of oppression contribute to higher rates of mental health conditions among girls and women (48). While this area is highly complex and many nuances exist, it appears that these patriarchal norms render girls and women more vulnerable to mental illness (49). Because of gendered norms related to mental illness, higher rates of benzodiazepines and other mood-altering pharmaceuticals are prescribed to women (50). The same is true for pain medications, making women susceptible to opioid addiction (51). In the US, between 1999 and 2016, women overdoses increased by 583%, compared to a 404% increase in men over the same time period (52).

Eating disorders and body image dysmorphia amongst BIWOC are based on dominant norms of beauty that are lodged within Eurocentric frameworks of thin body types with white skin (53). Skin bleaching is common among many BIWOC communities and particularly widespread within the South Asian diaspora. Brides with fair skin are highly prized within South Asian communities, even within Canada. Within some Southeast Asian communities, cosmetic surgery to increase the size of the eyelid is in fashion (54). Disordered eating within adolescent BIWOC communities is a serious concern, with 67% of Asian girls, 45% of Latinx girls, and 43% of Black girls reporting unhealthy weight control behaviors, in a large US study (55). That being stated, those BIWOC who identify with traditional cultural norms are protected from Eurocentric thin beauty ideals (56).

Mental health status is thus negatively impacted by racism and sexism. A reliance upon friends and family for mental health support is a barrier to treatment for some BIWOC (42). Furthermore, implicit biases within mental health treatment systems also influence BIWOC opportunities for healing and recovery. Negative stereotypes about BIWOC which may be subtle or even unconscious among mental health professionals, reduce the quality of mental healthcare for BIWOC (46).

### Physical health issues amongst BIWOC

Chronic exposure to stressors such as racism exacerbates weathering. This complex matrix of socioeconomic factors has been linked to poor sleeping patterns amongst BIWOC. Sleep is a foundational aspect of positive health status and is often ignored in health data, yet it bridges mental and physical health status and is thus an effective measure of population health trends. Sleep difficulties are usually linked to an overactive mind. Mental restlessness is typically rooted in stress. Traumatic events, microaggressions, sexualized violence, and other stressors often cause sleep disturbances (57, 58). Besides stress induced sleep concerns, BIPOC have higher rates of obstructive sleep apnea (OSA) compared to people of European heritage in the US. Most strikingly, Black children are diagnosed with OSA at a rate four to five times that of children of European heritage (59). Sleep disturbances and disorders are associated with many health problems, such as cardiovascular disease (CVD), diabetes, reproductive health, obesity, and so forth, all of which are worse for BIWOC than their counterparts of European heritage in the US (60).

Breast cancer survival rates for US BIWOC are significantly low—BIWOC under the age of 50 years are 127% more likely to die of breast cancer than women of European ancestry (61).

High rates of hypertension are found amongst BIWOC in North America, an unsurprising finding given that chronic stress often results in hypertension (62, 63). Black women, for example, are at high risk of stroke partly because of increasing hypertension severity (64).

Various factors relating to weathering, including high rates of maternal depression and anxiety contribute to high rates of morbidity and mortality during pregnancy, particularly for Black women in the US, whose chances of dying during childbirth are three to four times higher than women of European heritage (63). Preeclampsia, gestational diabetes, pre-term birth and low birth weight afflict BIWOC more than other women in the US (63).

Iron deficiency is a long term health problem for Latinx, Black, and South Asian women in North America (65, 66). Lack of iron leads to extreme chronic fatigue, anemia, pregnancy complications, heart problems, and other health issues (65, 67).

Health status is impacted mainly by socioeconomic status, but health systems and clinical care also have a significant impact on BIWOC mental and physical health. Implicit biases of HCWs lead to compromised healthcare in Canada and the US, resulting in higher readmission rates, greater risk of infection, and other problems that have left BIWOC dead from preventable conditions (68, 69).

All in all, more data, specifically analyzing BIWOC are urgently required in order to inform health promotion, disease prevention, treatment, rehabilitation, and palliation efforts. Current data paint a disturbing image of mental and physical health compromises as a result of chronic stressors and other aspects of weathering. Poverty, racism, misogyny, violence, lack of health information, and other factors are responsible for these concerns—all of which are preventable. Anti-racism and feminist approaches to health programming, designed and implemented by BIWOC, would go a long way toward improving this situation.

## Canadian BIPOC data collection during the pandemic

Indigenous communities live with COVID-19 at 6-fold higher rates than other Canadians, although they comprise only 5% of the population (70). Beyond higher death rates, 6 out of 10 Indigenous people report that the pandemic significantly worsened their mental health; yet, fewer Indigenous people reported that they have applied for federal income support (71). Within Indigenous communities, women, similar to other communities, provide most of the care to family members who are unwell with COVID-19. Besides this work inside the home, many Indigenous women also participate in the waged economy, as well as offering services as volunteers to support elders and others.

The City of Toronto Department of Public Health was the first jurisdiction to collect disaggregated data related to ethnocultural status. Rarely do Canadians hear that 38% of confirmed COVID-19 cases are BIWOC, while BIWOC represent 27% of Toronto’s racialized population (72).

**Figure 1:**
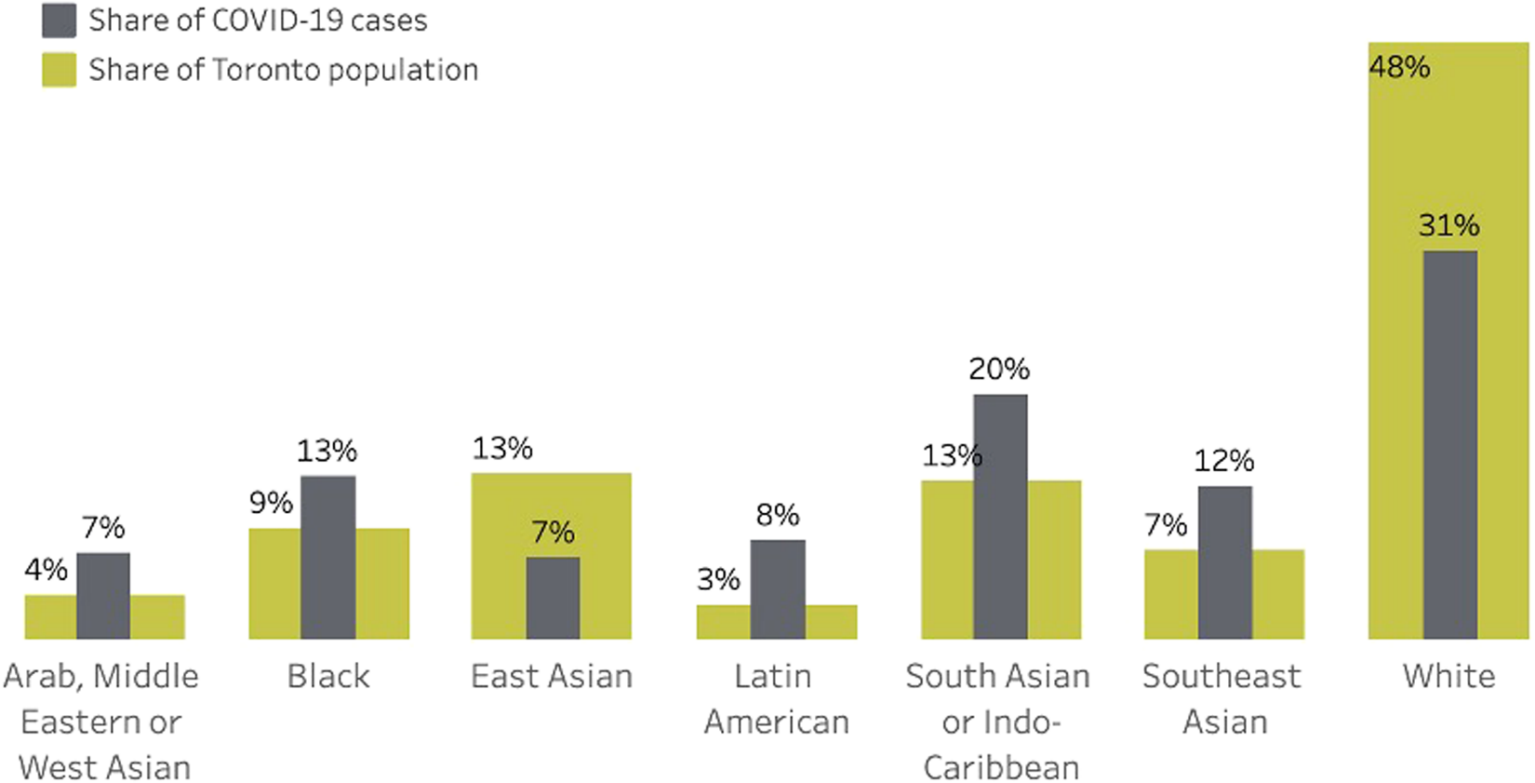
COVID-19 rates in Toronto, Ontario by ethnic group.

If data from the rest of the country continue this trend, then specific COVID-19 efforts ought to be focused on BIWOC. Figure 1 illustrates the disproportionate rates at which BIPOC are living with COVID-19 in Toronto, as of December 31, 2021 (*N* = 139,965). Data were collected from the City of Toronto’s public data portal.

## Racialized women and the pandemic

Much of women’s labor is unrecognized. It occurs inside the home, where up to 75% of unpaid duties are performed by women (73). The home is also the site of violence against women, which has doubled and tripled during the pandemic, globally (74), and is significantly worse for BIWOC, partly related to structurally-created poverty and unemployment, giving BIWOC fewer choices and escape routes (75).

Outside of the home, racialized women earn approximately 80% of racialized male earnings and approximately 57% of all male earnings in Canada; in 2015, only 13.8% of the top 1% of earners were BIWOC (76). Long term underfunding by the Canadian state of First Nations, Inuit and Metis services further undermine Indigenous women’s determinants of health, which then leads to higher risks of viral infections such as the coronavirus.

Racialized women’s jobs in retail, hospitality, and other pink-collar sectors generally put them at greater risk of being laid off in this recession. Racialized women in healthcare, on the other hand, are typically overworked, underpaid, and at increased risk of morbidity and mortality (77). Canada’s current healthcare crisis is similar to those around the world—HCWs are experiencing high rates of exhaustion, mental health conditions, and burnout (78).

## Care aides

Care aides (CAs) (or Personal Support Workers, Home Support Workers, nursing assistants or other occupational titles) represent a crystallization of health and medical care issues related to COVID-19. The majority of care aides are women, of whom approximately 47% are racialized women (79). Black and Filipino women filled the ranks of nurse aides, orderlies, and patient service associates in high numbers (80). In long-term care homes, CAs constitute approximately 90% of the bedside labor force (81). It appears that most women CAs in Canada are first-generation immigrants of colour or emanate from more settled racialized communities (82). It appears a significant portion of CAs also originate from Africa, South East Asia, the Caribbean, and elsewhere (83) and these numbers are projected to rise in North America (84).

Given the highly gendered and racialized nature of care aid work, understanding CAs and the nature of their working conditions is critical for the improvement of BIWOC health. CAs are considered to be at the bottom of the healthcare hierarchy; their jobs entail some of the work that other health professionals would prefer not to do, such as changing bed pans, bathing patients, lifting and other heavy tasks.

Prior to the pandemic, the work of CAs has been undervalued, despite shortages in the CA workforce, and the pandemic highlights this reality (85). Wages and working conditions across job sites are often challenging. Approximately one-quarter of the CA workforce needs to work at more than one job site to make ends meet or because they are unable to find full-time work (81).

### Elder care: long term care

To make matters more challenging, most CAs function within a healthcare infrastructure that falls outside the scope of public healthcare in Canada. Most of Canada’s healthcare system is public, guaranteeing access to all those who are eligible. On the other hand, care homes, one of the job sites for CAs, are available at varying rates and only some of which are covered by the public purse. Some care homes are not for profit, but those which are fully private charge upwards of $10,000 per month (86). Yet the privatized nature of such facilities does not offer the CAs and other non-unionized workers comparable luxury in their working conditions.

Partially because of the privatized nature of long term care facilities (LTFs), regulatory mechanisms, while they exist, are not always followed, resulting in shoddy labor standards and poor working conditions for staff. One of the most visible of these LTFs was the Lynne Valley Care Home, which before the pandemic, was well known as a facility with systemic inequities and poor treatment of staff (87).

Finally, to add more complexity, the context within long-term care facilities exists is primarily linked to the care of the elderly. In Canadian society, like in most individualist societies that value youthfulness, seniors typically lack respect. LTCs are a concentrated example of the poor treatment of elders in this society (88). Most residents in LTCs are women, partially because women live longer than men (89). In most age categories, more men are dying from COVID-19 than women except in the 80-year-plus category, in which more women are dying than men (90).

### Home care

In the US, the majority (87%) of approximately 3.5 million home care (HC) providers are women and most are people of color (62%) and immigrants (31%) (91). Within Canada, this is the work of CAs, personal support workers (PSWs), and related occupations. Before the pandemic, there was a great need for healthcare aides, which has only augmented migration from the Global South to the Global North, accentuating brain drain. Although HC aides are in high demand, they are poorly paid with an average income of about $25,000 USD, putting them below the poverty line. Some CAs work in clients’ homes, offering essential services for medication management, personal cleanliness, nutrition, mobility and so forth. Most HC workers provide care for clients over 65 years of age. During the pandemic, home support work has been critical in keeping elders outside of LTFs. The pandemic has also raised many challenges for HC—related to clients’ fears that they may spread the virus and concerns about bringing the virus home to their families. Home settings typically do not offer regulated environments where infection control procedures are in place. HC workers thus face numerous challenges in maintaining their health status during a pandemic which is spread through airborne vectors.

### Working conditions

According to a Canadian CA, “The working conditions are very difficult. You’re working short all the time, you’re never guaranteed registered nurses and often your only option is to send your patient to the hospital when often it’s not what they need and what’s best for them” (85). Working without sufficient staffing, CAs are often working to exhaustion. Their pay is between $19–$26 an hour (92), which is closer to meeting the threshold of the living wage of BC (93), as long as it is full time work. One of the positive developments during the pandemic was the raise in hourly wages for CAs in most parts of the country (94). Besides pay, benefits vary from employer to employer. Health authorities offer 18 days per year of sick leave in contrast to the 5–7 days per year that many CAs receive (85).

### Lack of employment insurance

Adding to this concern is the lack of employment insurance for some CAs (85). The picture is clear—CA experience poor working conditions within a privatized part of Canada’s otherwise universal healthcare system. As Estabrooks notes:

“[T]he pandemic did not cause the crisis; it came along and caused a massive shock to the long-term care system, shining a harsh light on fractures in a system that was ripe for catastrophe” (95).

### Caregiving is disrespected

The disrespect dealt to CAs points to the larger problem of disrespect to caregiving in general, a highly feminized issue. Care work is an extension of feminized labor practices and hierarchies within a system that places greater value on the work that is traditionally carried out by men.

Like bushfires, COVID-19 runs quickly through LTFs and significant conversations about the causes of this issue have been held nationally for many years, only intensifying and gaining widespread public attention during the pandemic. In many LTFs, CAs have been identified as a source of introducing the virus, which has been exacerbated by the pre-existing and underlying health issues of patients (96).

**Figure 2:**
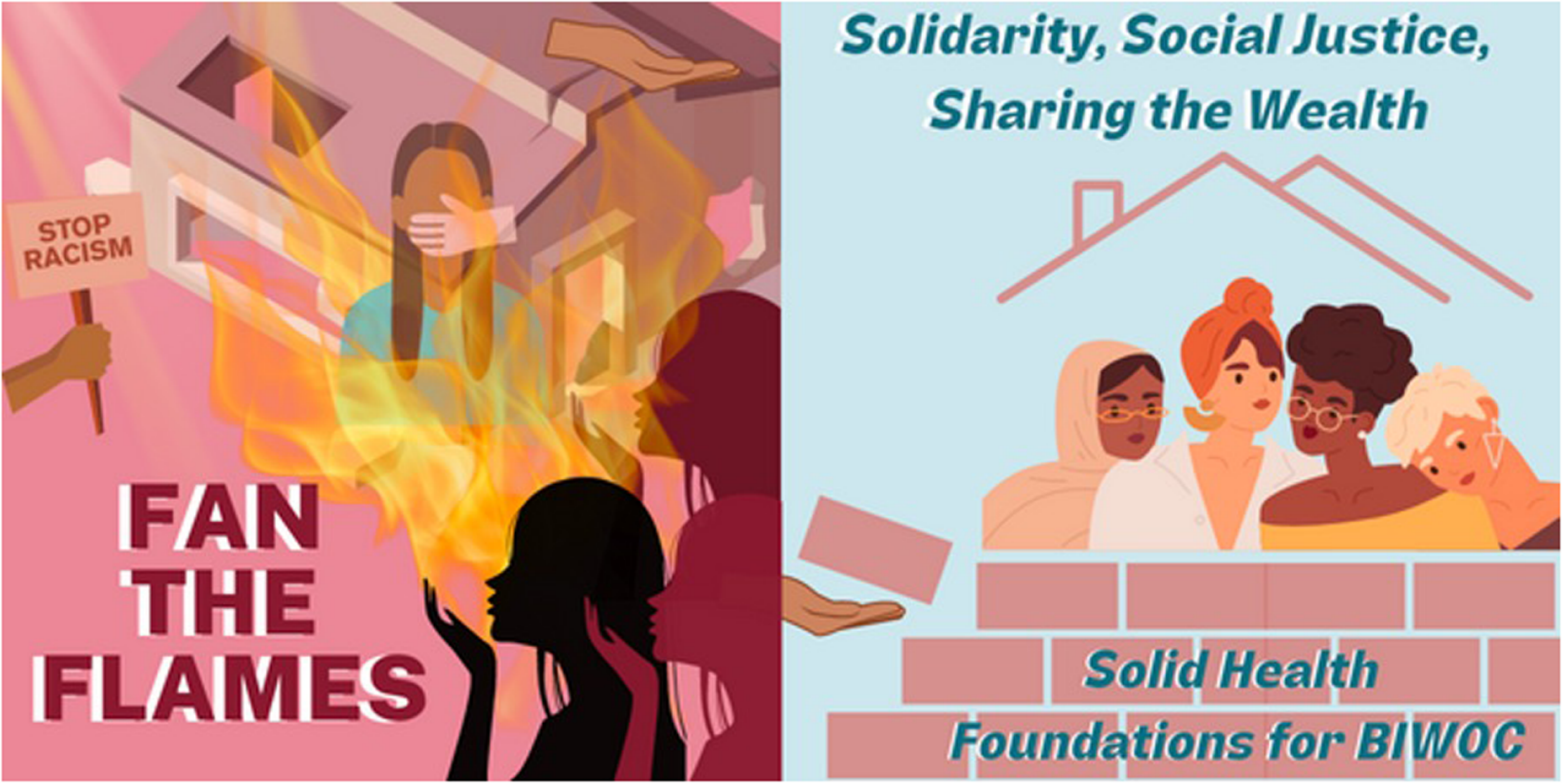
Fanning the flames to burn down structures of health inequity and rebuild structures that catalyze solidarity, social justice, and sharing the wealth (diagram created by Parisa Kabir and Brenda Ma, with creative input from Sheyn Hosanee and Jaya Kailley).

Canadian BIWOC, particularly those living in poverty or with disabilities or other intersecting inequities, struggle for their health beginning with virtually every social determinant of health. Burning down the structures that have created the conditions for these stark inequities is a first step towards addressing the urgent health crisis in this population. As seen in Figure 2, the next step is to create solid health foundations for BIWOC such that solidarity, social justice, and the true sharing of wealth in all its dimensions will occur.

## Recommendations to support better health for racialized women

The very edifice of institutional and social life ought to keep changing in order to promote better health for BIWOC. Building the bonfire of transformation takes time. Below are a few suggestions to move in that direction. These recommendations are inevitably incomplete. Others may be able to conceptualize different solutions to the problems of racism, sexism, and various forms of oppression experienced by BIWOC. I offer these non-exhaustive recommendations as a contribution to the dialogue that started decades ago and will hopefully continue.

### Hiring more racialized women

In the current climate, it has become *flavor of the month* for organizations and large institutions to declare they support Black Lives Matter and other such groups, thereby implying they are not racist. Moving beyond rhetoric, however, is critical.

Hiring more racialized women in groups of twos, threes or more, in well-paid and secure positions, will go a long way to addressing systemic inequities. York University, McMaster University, Simon Fraser University, University of Alberta, and others are hiring several new Black faculty members. Making genuine efforts like these, to correct historical wrongs, are illustrative of institutions that are genuinely interested in long-term systemic change. Institutions in healthcare, education, forestry, and other private and public institutions ought to follow suit.

It is critical that initiatives like these continue and are not one-off events. Furthermore, decolonizing targeted hirings will make a long term positive impact. By hiring BIWOC who have a strong sense of anti-racism and decolonial realities and will work with other racialized peoples to support collective well-being, a strong emphasis on addressing the *colonized mind* becomes a focus. Hiring those who embrace solidarity and support for other BIWOC is key to counteracting prevailing *divide and conquer politics* and racialized norms which have created large numbers of BIWOC who internalize racism and may be labelled as *coconuts*, *apples*, *Oreos*, or *bananas* and others who are darker on the outside and white on the inside.

Furthermore, cultural change within institutions is key to providing safe environments for racialized women. Employment equity is good for the bottom line as a 1% increase in workplace ethnocultural diversity leads to a 2.4% increase in revenue and a 0.5% increase in workplace productivity (97).

### Social determinants of health (SDoH)–honoring all women’s work, improving food security, internet access and more

The financial impacts of COVID-19 are disproportionately experienced by Canadians, with all racialized groups reporting higher levels of hardship. Given the pre-existing higher poverty rates among BIPOC Canadians, work disruptions have made life even harder.

Working from home has provided a better work-life balance for some women despite its challenges. Some women, particularly those in white-collar positions, have benefited from saving time commuting and being able to devote more attention to family, self-care, gardening, and other activities. Given perennial and ubiquitous challenges with finding safe, reliable, and affordable childcare, the pandemic has helped some families by bringing them together for more time–a silver lining of the pandemic (98).

Legislation and/or policies that would benefit women include those that support long-term options for work that allows for a sustainable pace of work, flexible hours, and options for regular cybercommuting. Performance reviews that take into account the disruptions to women’s careers are also critical. Given current conditions, better unemployment insurance options are also key (98).

Hiring practices such as targeting more racialized women will also produce “positive outcomes for organizations with respect to communications, negotiations, structure and authority” (99). Training to address unconscious bias in the workplace is another step. Even with equitable laws and policies in place, people still unconsciously hold involuntary prejudices and biases against, most often, racialized people and women (100), so this is a longer-term solution. It can be done through feminist anti-racism training by experienced facilitators with lived experience and would go a long way to making workplaces more suitable for women, particularly BIWOC.

Empathetic communications and fostering workplace cultures that respect women and racialized communities would also be game changers in Canada. Childcare on-site, breastfeeding stations, and other workplace resources to support mothers’ needs, will address vital labor force concerns.

Governments, businesses, and other agencies could provide early childhood development resources, implement policies to reduce childhood poverty, provide work and income support opportunities for adults, and ensure healthy housing and neighborhood conditions. Improving food security would make a big difference to family health. The most powerful mechanism to improve food security is income enhancement, either through living wages for all and/or guaranteed basic income (GBI). Other initiatives include alternatives to food banks, such as food pantries that mirror neighborhood little free libraries, in which people deposit non-perishable food and others take food as and when required. Community gardens, fruit-tree projects, community kitchens, and other effective initiatives ought to be expanded.

More suitable housing will meet fundamental needs for shelter security—a critical determinant of health. Expanding co-op housing and co-housing are two viable models that have been tried and tested within Canada (101). The provision of more safe houses and shelters for women would meet an exigent need, as increased domestic violence against women during the pandemic has further exposed gaps in service provision.

Moreover, current efforts to provide affordable broadband internet to inner city communities, which are rural and remote, will help to bridge the digital divide in Canada. This will make a particularly big difference for low income BIWOC.

### Improving the healthcare system

Addressing racism against BIPOC communities within the healthcare system is an exigent need. Mandatory widespread training programs that foster cultural safety for all racialized people is one possible solution. Following up these programs with onsite discussions in each workplace would help to address the gap between theory and practice. Significant resources are required to address these serious issues.

Schools of medicine, nursing, physiotherapy, midwifery, and other health professions ought to increase admission rates for racialized students, particularly Black and Indigenous students. This will help to create a healthcare workforce to better reflect the population it serves. Moreover, improving fairness within credentialing systems for foreign-trained HCWs would meet critical shortages in the Canadian health system, create a more diverse healthcare workforce, as well as promote the full potential of immigrants who are already in Canada.

Integrating traditional systems of medicine would further assist BIPOC patients to experience ancestral and cultural forms of care. Traditional healers are slowly integrating into the dominant system of healthcare. This holds promise for attending to emotional, spiritual, and physical health needs (102, 103).

### Data collection and community-based programming

More granular data collection related to COVID-19 cases and deaths, focusing on ethnocultural status, would help Canada to have a better picture of who is living and dying with this disease. As noted in the Grandmother Perspective Report, these data need to be protected through legislation such as an Anti-Discrimination Data Act to regulate the use of demographic data in conjunction with the human rights and information and privacy concerns.

Furthermore, taking Toronto and Ontario’s lead, and learning from their lessons will be prudent for the rest of the country. Once more robust data related to communities of color are available, then community-driven programs, resourced by governments, ought to be put in place. In the West, Fraser Health has identified a need to start doing specific programming for South Asian Canadians based on their knowledge that the pandemic is impacting this community in a disproportionate manner (104).

Data collection alone will not solve the problem. Action is much more crucial, particularly if it is driven by affected communities. Community-based and community-driven programming holds great promise for solving the problems of racialized communities. Canada has a track record of working with communities of color to use data to drive community-based programming, which was well illustrated in HIV programming during the late 1990s and early 2000s.

### Promoting vaccine equity through prioritizing the needs of racialized communities

Some remote, rural, and Indigenous communities were at the top of the list for receiving COVID-19 vaccines in Canada. While the roll-out took longer than anticipated, approximately 81% of First Nations people received one dose (105).

Likewise, organizations which represent communities of color around the country called for the prioritization of the vaccine roll-out for communities that were hardest hit by the pandemic, namely Black communities and other racialized communities. In Toronto, where data clearly highlighted that Black communities were disproportionately impacted, Dr. Akwatu Khenti and organizations such as the Black Health Alliance were advocating for their community members to be at the top of the list for the vaccine roll-out. By 2022, 82% of the Black community who wanted the vaccine were vaccinated (105).

Similarly, various groups representing South Asians in Canada, such as The South Asian Health Research Hub and The South Asian COVID Task Force were calling upon governments to meet the needs of South Asian community members who were vulnerable, partially due to higher rates of diabetes and cardiovascular disease, a potentially deadly combination. Many South Asians in Canada also work in precarious or front-line jobs. By 2022, 96% of the South Asian community who wanted the vaccine were vaccinated (105).

A common mistake amongst researchers, journalists, and others was to conflate vaccine hesitancy with BIPOC communities’ mistrust of the Canadian healthcare system. Vaccine hesitancy typically, although not always, emanates from anti-science, anti-authoritarian, and pro-religious perspectives that overlap with anti-mask ideology (106, 107). While some racialized people belong to these groups, it is critical to distinguish the reasons why some BIPOC were trepidacious about trusting new vaccines. Caution, backed up by sound evidence and experience, was a savvy anti-racism approach. To be clear, there is no proof that BIPOC, in general, holds views that would belong to an anti-science viewpoint. Research with BIPOC communities in Canada illustrates that amongst those who held off from immediate vaccination, logistics such as long distances and difficulties with securing appointment time, accounted for lower vaccine uptake (108). Furthermore, some BIPOC were unable to be vaccinated because of a lack of access to online technologies, cold chain medical equipment, language barriers, and lack of identification documentation (108, 109). Finally, other BIPOC felt they needed more information about COVID-19 and the science behind vaccines (108).

All in all, by 2022, 83% of Canadians were vaccinated (110). All Canadians who wished to be vaccinated were eventually able to do so (111). Many BIPOC and other Canadians were concerned about vaccine hoarding and would have preferred the Canadian government to send more vaccines to the Global South (108).

## Prioritizing research on BIWOC

Biases within the research industry in this country have resulted in lower success rates for women researchers in competitive grant applications, compared to male researchers’ applications (112). This includes racialized women researchers. Given that some racialized women researchers are motivated to improve conditions within their own communities, the lack of funding is a serious barrier.

Looking at the data provided by The Social Science and Humanities Research Council (SSHRC) in Canada, for example, 56% of applicants were women, 19% of applicants were racialized people and only 3% were Indigenous people. On a celebratory note, 60% of the awardees were women who received 53% of the funding (113). None of Canada’s main research bodies include information about BIWOC research and researchers—an error which could easily be rectified.

Most Canadian researchers are entrepreneurial individuals who will *follow the money*. Once research bodies provide financial incentives for studies pertaining to the lives and health of racialized women, this research promises to bloom. The University of Toronto’s new Master of Public Health (MPH) in Black Health will inevitably help health research on Black women to blossom.

## Parting thoughts

Racialized women’s health needs ought to be a higher priority for governments, researchers and healthcare systems. Currently, racialized women’s morbidity and mortality rates, in a variety of categories, demonstrate compromised mental and physical health status, which is linked to lower income levels, racism, sexism, and other structural disparities. Less access to health and social services and mistreatment within these services also play a role.

The mental and physical health impacts of racism on health status include weathering, which has an impact on depression, hypertension, and shorter life spans (8). To make matters worse, healthcare systems have been criticized for their prejudiced treatment of BIPOC communities for decades (10).

During the pandemic, women’s unpaid labor—cooking, cleaning, caring, etc.—has increased significantly, creating more fatigue, mental health concerns, and in some cases, lost wages (114). Being forced to stay at home has substantially increased violence against women all around the world, starting in Wuhan, China where rates doubled (74). In Canada, rates of death and violence against BIWOC are higher than those of other women, partly related to higher unemployment and poverty rates, which diminish escape routes and alternatives (75). Indigenous women’s domestic homicide rates, in particular, are twice that of other Canadian women (115).

Although not new, the murders of BIPOC sparked a bigger bonfire than in the past. This time there is a strong possibility that this bonfire will burn down the house, meaning that the foundations of dominant institutions will be razed, to construct more equitable structures.

City of Toronto public health data paint a disturbing picture of health during the COVID-19 pandemic: 69% of female COVID-19 cases are BIWOC, who represent 54% of the total female population in Toronto (116). Data collection related to ethnocultural communities ought to be carried out in other parts of Canada, as long as these data will be first in many steps of community-driven solutions to health problems.

One of the most impactful ways to improve the health of BIWOC is through employment equity measures, such as hiring women in groups of two or higher. This is a win-win situation as productivity and profits increase with greater diversity. Improving food security, internet access, the conditions of women’s work, and prioritizing research on BIWOC also promises to improve population health status for BIWOC.

Ensuring that all women’s basic income, childcare, housing, food and water security, and other needs are met—will improve the health of children, older generations, and those who rely on women. Uplifting women’s human rights, social determinants of health, and care needs is a proven way to lift up entire communities and ultimately, nations. Paying particular attention to the needs of racialized women in societies like those in Canada promises to be a game changer.
